# Changes of Regulatory T and B Cells in Patients with Papillary Thyroid Carcinoma after ^131^I Radioablation: A Preliminary Study

**DOI:** 10.1155/2013/683768

**Published:** 2013-10-08

**Authors:** Lei Jiang, Yanxia Zhan, Yusen Gu, Yi Ye, Yunfeng Cheng, Hongcheng Shi

**Affiliations:** ^1^Department of Nuclear Medicine, Zhongshan Hospital, Fudan University, 180 Fenglin Road, Shanghai 200032, China; ^2^Department of Hematology, Zhongshan Hospital, Fudan University, 180 Fenglin Road, Shanghai 200032, China; ^3^Biomedical Research Center, Zhongshan Hospital, Fudan University, Shanghai 200032, China

## Abstract

*Introduction*. Lymphocytic infiltration and specific lymphocytes subsets may play important roles in papillary thyroid carcinoma (PTC) progression and prognosis. In this study, we try to understand the influence of ^131^I radioablation on the important lymphocytes subtypes of regulatory T and B cells (Tregs and Bregs). *Methods*. Peripheral blood mononuclear cells from 30 PTC patients before and after ^131^I therapy, and 20 healthy donors were collected. The expression of Tregs (CD4^+^CD25^+^CD127^−/low^) and B cell (CD5^+^CD19^+^) and production and secretion of interleukin 10 (IL-10) were analyzed by FACS and ELISA assay, respectively. *Results*. For Tregs percentage in peripheral blood lymphocytes, there was no difference between pretreatment and control and between posttreatment and control. Compared with pretherapy, increased Tregs infiltration was noted in posttherapy (*P* < 0.05). Although no difference was between pretreatment and control, compared with these two groups, decreased CD19^+^ and CD5^+^CD19^+^ B cell percentage in posttreatment was observed (*P* < 0.05). Among these groups, no significant difference was displayed in intracellular IL-10 production and extracellular IL-10 secretion. *Conclusions*. ^131^I Radioablation increased Tregs and decreased CD19^+^ and CD5^+^CD19^+^ B cells percentage after treatment. However, it has no effect on IL-10 and lymphocytes in peripheral blood. Therefore, longer follow-up of Tregs and Bregs should be further investigated.

## 1. Introduction

Thyroid cancer is the most common endocrine malignancy, which constitutes approximately 1% of all human malignancies [1, 2]. Papillary thyroid carcinoma (PTC) accounts for about 70% of all thyroid carcinomas [3]. Although it has a relatively good prognosis, the incidence of thyroid carcinomas is rapidly increasing, and 10–30% of patients have recurrence and/or metastases [4, 5].

At present, many mechanisms are involved in the development of thyroid cancer, and the dysfunction of the immune systems is increasing being considered [6]. Regulatory T cells (Tregs) are subtypes of CD4^+^ T cells, which play an important role in the immune response [7, 8]. In general, Tregs is identified as CD4^+^CD25^+^CD127^−/low^ or CD4^+^CD25^+^Foxp3^+^ [9, 10], which are commonly rich in primary tumors, draining lymph nodes, and peripheral blood of cancer patients [11–16]. An increased frequency of Tregs has been proven to be related with poor prognosis of many tumors [3, 17–19]. In contrast to Tregs, regulatory B cells (Bregs) mainly suppress immune response via the production of interleukin 10 (IL-10) [20, 21]. However, there are no specific transcriptions or surface molecular makers identified as Bregs. The most well-established concept of Bregs is the subtype of B cells producing IL-10, which are regulatory B10 cells. Furthermore, lots of studies demonstrated that the surface membrane marker CD5 was expressed on B10 cells. Meantime, CD19 is the well-established marker to identify B cells from peripheral blood mononuclear cells (PBMCs). Thus, it seems that the expression of CD5^+^CD19^+^ is a common feature or hallmark of regulatory B cells [20–23].

In clinic, large studies have proven that, compared with thyroidectomy alone, the combination of ^131^I radioablation and thyroidectomy for PTC patients could reduce recurrence and/or metastases [24–26]. In addition to that, the previous study demonstrated that Tregs and IL-10 level are related with invasiveness and prognosis of thyroid cancer [3, 5, 27, 28]. Therefore, it is necessary to understand the role of specific lymphocytes subsets in PTC patients before and after ^131^I radiotherapy to evaluate the relationship between immune response and ^131^I ablation. 

## 2. Materials and Methods

### 2.1. Patients and Controls

From October 2012 to December 2012, PTC patients, with total or near-total thyroidectomy, were admitted and carried out the first radioiodine ablation in the Department of Nuclear Medicine, Zhongshan Hospital. On the basis of laboratory tests and imaging modalities (ultrasound, CT, whole body ^131^I scan, and ^18^F-FDG PET/CT examinations, etc.), the patients with thyroiditis and/or long distant metastases (such as lung and bone) were excluded. The follow-up examination was one month after the 1st ^131^I therapy, and 30 patients had serum thymoglobulin level less than 2 *μ*IU/mL (normal: 0.27–4.2 *μ*IU/mL), that is to say, the ^131^I therapy was successful, were enrolled in this study. Moreover, the healthy control group consisted of 20 adult volunteers. 

The study was approved by local Medical Ethics Committees of Zhongshan Hospital, Fudan University. Informed consents were signed by all the patients before they were included in the study.

### 2.2. Radioiodine Ablation

In this study, all patients were firstly administrated for radioiodine ablation, and the received dose was 3,700 MBq (100 mCi) (Shanghai GMS Pharmaceutical Co., Ltd.). The venous blood samples of patients were collected within one week before the radioiodine therapy and one month after treatment, respectively. Meanwhile, the thyroid function tests and routine hematological parameters were analyzed.

### 2.3. Isolation of PBMCs

Venous blood samples were collected into ethylenediaminetetraacetic acid-treated tubes and diluted at 1 : 2 with Hanks balanced salt solution (HBSS) before Ficoll-Hypaque gradient centrifugation (2,000 rpm at room temperature for 15 min). Washed and resuspended, PBMCs were cryopreserved in fetal bovine serum containing 10% dimethyl sulfoxide (DMSO) and stored in liquid nitrogen for future cell-surface staining and cell culture.

### 2.4. Cell Culture

Cryopreserved PBMCs were thawed at 37°C and washed twice with HBSS. Then, PBMCs were seeded at a density of 5 × 10^5^/mL in 24-well tissue culture plates. The culture medium was RPMI 1640 medium supplemented with 10% heat-inactivated fetal bovine serum, 2 mM L-glutamine, 200 U/mL penicillin, and 100 *μ*g/mL streptomycin. For intracellular staining of IL-10, cells were stimulated with 50 ng/mL phorbol-12-myristate-13-acetate (PMA) and 500 ng/mL ionomycin for 24 h and the additional presence of 1 mM Brefeldin A (BFA) for last 4 h. 

### 2.5. Flow Cytometric Analysis of Tregs

~2 × 10^5^ PBMCs were incubated with FITC-conjugated anti-CD4/PE-Cy7-conjugated anti-CD25/APC-conjugated anti-CD127, or isotypes (BD, USA) for 30 min at 4°C, washed twice and resuspended in staining buffer for analysis of T cell subpopulations. Acquisitions were performed on a FACS Aria II flow cytometer (BD, USA) and then analyzed using Flowjo software version 7.6.

### 2.6. Flow Cytometric Analysis of CD5, CD19, and IL-10

The flow cytometry was performed as above described. Briefly, ~2 × 10^5^ PBMCs were incubated with APC-conjugated anti-CD5/FITC-conjugated anti-CD19 or isotypes (BD, USA) for 20 min at 4°C, washed twice, and resuspended in staining buffer for analysis of B cell subpopulations. 

For intracellular IL-10 detection, cultured cells were then fixed and permeabilized before PE-conjugated anti-IL-10 or isotype (BioLegend, USA) staining. Acquisitions were performed on a FACSAria II flow cytometer (BD, USA) and then analyzed using Flowjo software version 7.6.

### 2.7. IL-10 Enzyme-Linked Immunosorbent Assay (ELISA)

According to the manufacturer's instructions of Human IL-10 Quantikine ELISA kits (R&D, USA), concentration of IL-10 in supernatants of cell culture was detected. The lower detection limit of this assay was 0.78 pg/mL. Pure RPMI 1640 medium was used as negative control.

### 2.8. Statistical Analysis

SPSS 18.0 software for Windows (SPSS Inc., Chicago, Iee, USA) was used for statistical analysis. Data were expressed as mean ± SD. Means were compared using the Student's *t*-test. When multiple groups were compared, one-way ANOVA and Kruskal-Wallis test were used for data fulfilling normal distribution and for those did not, respectively. A 95% confidence level was chosen to determine the significance between groups, with *P* values of less than 0.05 indicating significant differences.

## 3. Results

### 3.1. Clinical Data

As shown in Table 1, 30 PTC patients were enrolled in this study, including 20 females and 10 males, with age range 26–70 years, median 45 years. Twenty healthy donors were also included, consisted of 11 females and 9 males, with age range 22–63 years, median 43 years. 

The absolute number of lymphocytes in the pretreatment ([2.45 ± 0.68] × 10^9^/L) and posttreatment group ([1.52 ± 0.41] × 10^9^/L) is within normal levels (1.1–3.2 × 10^9^/L). However, there is significant difference between the pretreatment and posttreatment group (*P* < 0.05).

### 3.2. CD4^+^ T Cells and Tregs Screening by Flow Cytometry Analysis

**Figure 1:**
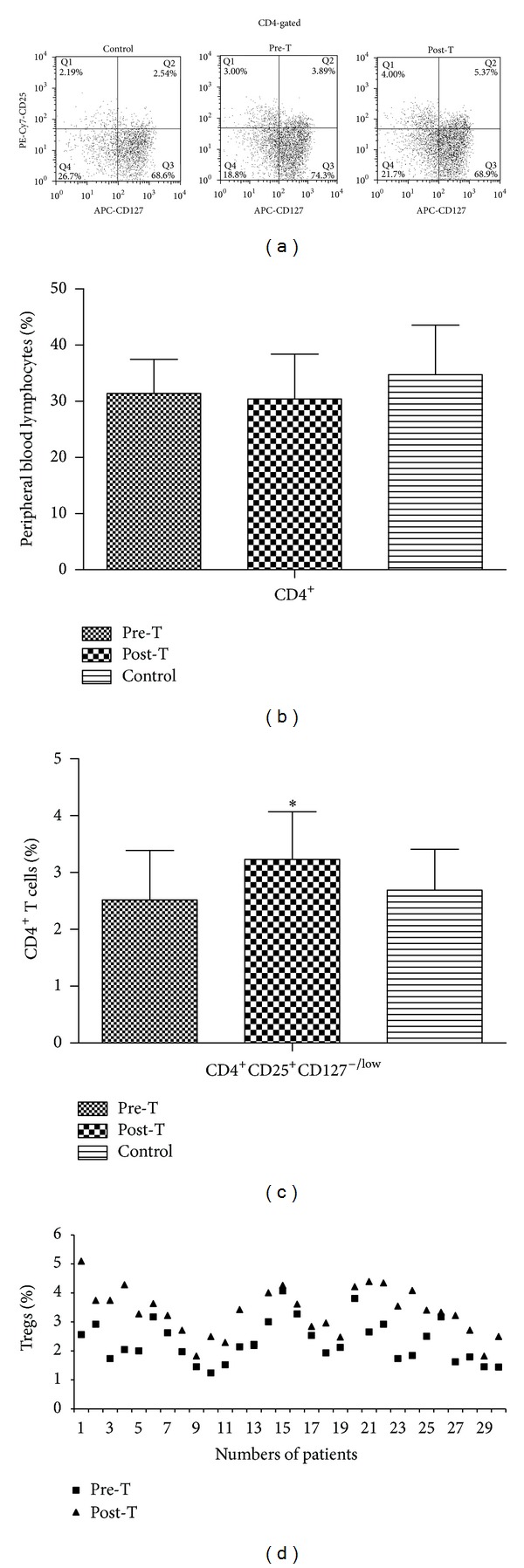
Expression of CD4^+^CD25^+^CD127^−/low^ T cells (Tregs) in blood sample of pretreatment, posttreatment, and control groups (Pre-T: pretreatment, Post-T: posttreatment). (a) CD4^+^CD25^+^CD127^−/low^ lymphocytes shown by FACS. (b) CD4^+^ T cells percentage in peripheral blood lymphocytes. (c) CD4^+^CD25^+^CD127^−/low^ T cells percentage in CD4^+^ T cells (*P < 0.05, compared with Pre-T). (d) Comparison of peripheral blood Tregs in individual patients with thyroid papillary carcinoma before and after ^131^I radioablation.

Blood samples of PTC patients and the healthy donors were analyzed by flow cytometry for CD4^+^ T cells and CD4^+^CD25^+^CD127^−/low^ T cells (Tregs), respectively (Figure 1(a)). The CD4^+^ percentage in peripheral blood lymphocytes of the pretreatment, posttreatment, and control groups was (31.40 ± 6.09)%, (30.38 ± 8.00)%, and (34.74 ± 8.84)%, respectively. There was no significant difference among the above groups (*P* > 0.05) (Figure 1(b)). 

Compared with Tregs (expressed as percentage of CD4^+^ T cells) in blood of PTC patients before ^131^I therapy (2.52 ± 0.87)%, Tregs was significantly higher in the posttherapy group (3.23 ± 0.84)% (*P* < 0.05) (Figures 1(c) and 1(d)). Tregs in the healthy donor was (2.69 ± 0.72)%. However, no significant difference was noted between the pretreatment and control group and between posttreatment and control, respectively (*P* > 0.05) (Figure 1(c)).

### 3.3. CD19^+^ and CD5^+^CD19^+^ B Cells Screening by Flow Cytometry Analysis

**Figure 2:**
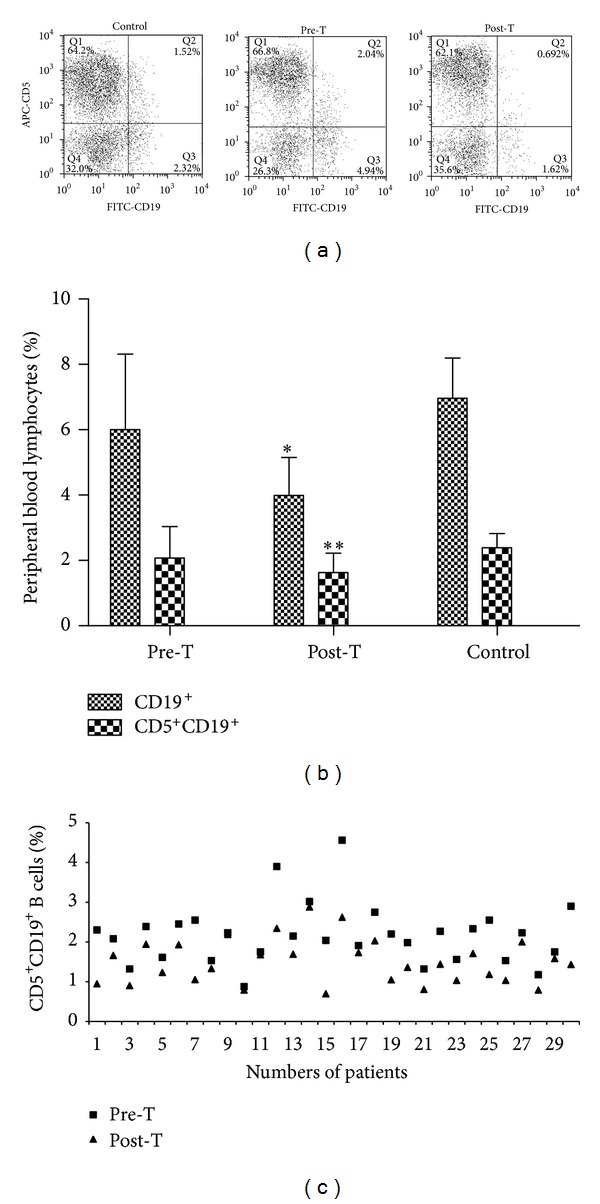
Expression of CD5^+^CD19^+^ B cells in blood sample of pretreatment, posttreatment, and control groups. (a) CD5^+^CD19^+^ B cells shown by FACS. (b) CD19^+^ and CD5^+^CD19^+^ B cells percentage in peripheral blood lymphocytes (*P < 0.05, compared with Pre-T and control; **P < 0.05, compared with Pre-T and control). (c) Comparison of peripheral blood CD5^+^CD19^+^ B cells in individual patients with thyroid papillary carcinoma before and after ^131^I radioablation.

Blood samples of patients with PTC and the healthy donors were analyzed by flow cytometry for CD19^+^ and CD5^+^CD19^+^ B cells, respectively (Figure 2(a)). The CD19^+^ percentage of total peripheral blood lymphocytes among the pretreatment, posttreatment, and control groups was (6.00 ± 2.31)%, (3.99 ± 1.16)%, and (6.96 ± 1.24)%, respectively. 

No significant difference was found between pretherapy and control (*P* > 0.05), but the significant difference was noted between posttreatment and control (*P* < 0.05) (Figure 2(b)). Compared with CD5^+^CD19^+^ B cells (expressed as percentage of total lymphocytes) in blood of PTC patients before the ^131^I ablation (2.07 ± 0.97)%, CD5^+^CD19^+^ B cells were significantly lower in the posttreatment group (1.63 ± 0.59)% (*P* < 0.05). CD5^+^CD19^+^ B cells in the healthy donors were (2.39 ± 0.43)%. Similar to the results of CD19^+^ B cells, there was no significant difference between pretreatment and control (*P* > 0.05), but the significant difference was observed between posttreatment and control, respectively (*P* < 0.05) (Figures 2(b) and 2(c)).

### 3.4. Accumulated Intracellular IL-10 in CD5^+^CD19^+^ B Cells by Flow Cytometry Analysis

**Figure 3:**
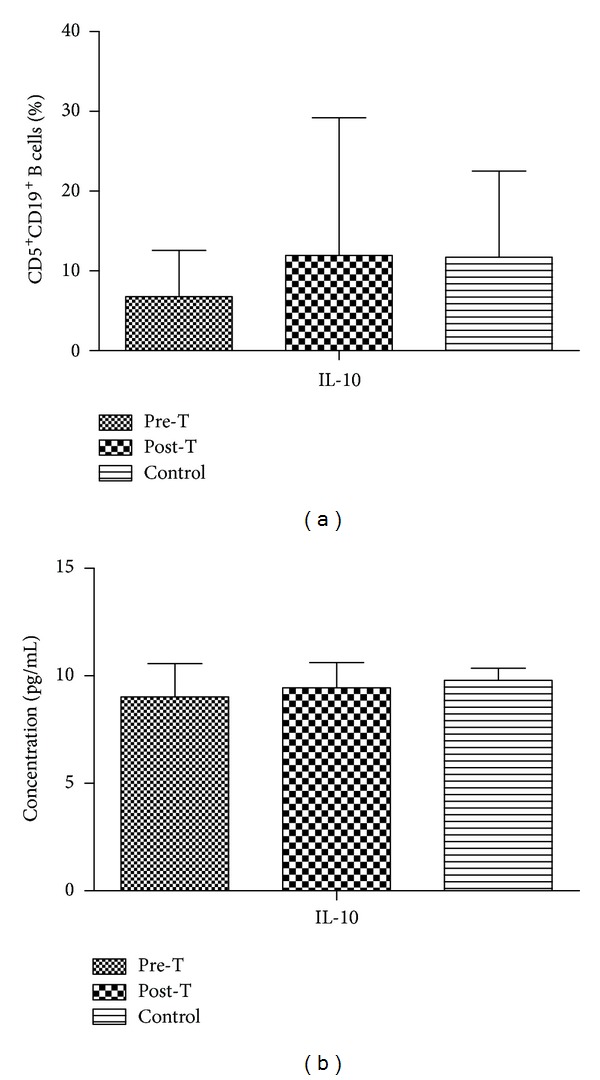
Intracellular and extracellular IL-10 in CD5^+^CD19^+^ B cells. (a) Intracellular IL-10 analyzed by FACS. (b) Extracellular IL-10 concentration tested by ELISA.

The intracellular production of IL-10 in CD5^+^CD19^+^ B cell percentage by flow cytometry analysis in the pretreatment, posttreatment, and the healthy donors was (6.77 ± 5.80)%, (11.96 ± 17.25)%, and (11.72 ± 10.81)%, respectively. There was no significant difference among these groups (*P* > 0.05) (Figure 3(a)). 

### 3.5. IL-10 Production by CD5^+^ B Cells by ELISA Analysis

The IL-10 production by CD5^+^ B cells by ELISA analysis in the pretreatment, posttreatment, and the healthy donors was 9.02 ± 1.55, 9.44 ± 1.18, and 9.79 ± 0.57 pg/mL, respectively. There was no significant difference among these groups (*P* > 0.05) (Figure 3(b)). 

## 4. Discussion

The lymphocytic infiltration is frequently observed in PTC, and specific lymphocytes subsets may be the important regulators of PTC progression and prognosis [3, 5, 27, 28]. Therefore, in this work, lymphocytes subpopulations of Tregs and CD5^+^ B cells (putative B cells that have regulatory functions) are studies in blood samples of PTC patient before and after ^131^I ablation. To our knowledge, no previous study focused on this control research.

Although Tregs have been studied and analyzed in many tumors, such as breast cancer, pancreas cancer, and melanoma [17–19], the studies on Tregs infiltration in thyroid tissues and peripheral blood samples are limited. Gogali et al. [3] and French et al. [5] proved that increased Tregs infiltration in thyroid tissue was positively correlated with advanced disease stage. In addition, Gogali et al. [3] showed that there was no difference in Tregs percentage in blood samples between PTC patients and the healthy control. Different from the patients included in previous studies [3, 5, 28], the population in the present study was PTC patients with total or near-total thyroidectomy, whose Tregs percentage in the peripheral blood samples was supposed to be within normal level [29]. The pretreatment Tregs in our study were consistent with previous studies. Compared with pretreatment, Tregs after the ^131^I treatment were significantly higher. However, the posttreatment Tregs percentage in peripheral blood lymphocytes was found to have no significant difference with the healthy donors. The main function of human immune system is the body protection from a diverse range of agents, including tumor cells and radionuclide [3]. Elevating Tregs may be the immune response to ^131^I, which was not beyond the self-tolerant extent and normalized the numbers of CD4^+^ T cells. Moreover, it is demonstrated that ^131^I ablation for PTC patients was a safe and effective therapy. 

In addition to Tregs, certain B cell subpopulations could also exhibit potential regulation of immune response by functioning as cellular adjuvants for CD4^+^ T cell activation and are involved in immune pathology through the production of cytokines that regulate T-cell function [30–33]. Given that regulatory B-cell subsets are likely to exist, one of the factors produced by regulatory B cells is the immunosuppressive cytokine IL-10 [20–23]. In this work, there was no significant difference between the pretreatment and control of CD19^+^ B cells and between the pretreatment and control of CD5^+^CD19^+^ B cells. However, compared with healthy donors and PTC patients before ^131^I ablation, both CD19^+^ and CD5^+^CD19^+^ B cells after the ^131^I therapy were significantly decreasing. The reasons are probably as followed: compared with T cells, B cells are more sensitive to radiation and prone to radiation-induced inhibition [34]. Moreover, although some clinical observations supported that the increasing of B cells was contributed to the limitation of autoimmune and malignant diseases, they are testified to have rather aggravating potential in other cases [35, 36]. Our results also showed there was no obvious correlation between decreasing B cells and bad prognosis. In addition, compared with the pretreatment group, the absolute number of lymphocytes in the posttreatment group significantly decreased, which was consistent with the analysis of regulatory B cells. We assume that in patients with thyroid papillary carcinoma before and after ^131^I radioablation, regulatory B cells prior to regulatory T cells play more important role in the regulation of peripheral blood lymphocytes.

IL-10 was initially associated with Th2 cells and was described to inhibit Th1 cytokine production [37–39]. However, at present, IL-10 is not only involved in the inhibition of Th1 polarization but also prevents Th2 responses and exerts anti-inflammatory and suppressive effects on most hematopoietic cells. IL-10 produced by monocytes and cells other than T cells is required to maintain Treg-suppressive function and other autoimmune diseases [40]. In our study, the intracellular and extracellular IL-10 production and secretion were found to have no significant difference between the pretreatment, posttreatment, and control groups, which are in accordance with results of CD4^+^ T cells and Tregs. However, the numbers of posttreatment CD19^+^ and CD5^+^CD19^+^ B cells were lower than those of pretreatment, which suggests that the synthesis and secretion of IL-10 were relatively increased. It could develop and maintain that posttherapy Tregs were higher than pretherapy ones in this study.

Moreover, there were some potential limitations in this study. One is considering the clinical effect of ^131^I, only one time-point after treatment was chosen, which couldn't reflect the dynamic change in patients' peripheral blood lymphocytes. Another limitation is the small population. Large sample of analysis of regulatory T and B cells in patients with thyroid papillary carcinoma before and after ^131^I radioablation should be further investigated. 

## 5. Conclusion 

^131^I radioablation increased Tregs percentage and decreased CD19^+^ and CD5^+^CD19^+^ B cells percentage at one month after treatment. However, it has no effect on the production and secretion of IL-10, and the percentage and absolute number of lymphocytes in peripheral blood are within normal levels. Therefore, longer follow-up of changes of regulatory T and B cells should be further investigated.

## Figures and Tables

**Table 1:** Clinical data of patients and healthy donors.

Variable	Patients		Healthy donors
	Pre-T	Post-T	
Gender (female/male)	20/10		11/9
Age (median, range, yr)	45 (26−70)		43 (22−63)
Free T3 (normal: 2.8–7.1 pmoL/L)	1.38 ± 0.77	5.31 ± 2.87	4.54 ± 1.60
Free T4 (normal: 12.0–22.0 pmoL/L)	2.60 ± 0.85	19.1 ± 13.1	16.72 ± 2.67
TSH (normal: 0.27–4.2 *μ*IU/mL)	84.56 ± 17.11	2.39 ± 2.73	2.14 ± 1.13
Tg (normal: 1.4–78 ng/mL)	13.32 ± 14.2	0.73 ± 0.65	22.72 ± 17.46
Anti-Tg (normal: <115 IU/mL)	15.36 ± 4.72	12.94 ± 4.10	20.82 ± 11.47
Anti-TPO (normal: <34 IU/mL)	10.99 ± 5.45	10.00 ± 5.27	8.86 ± 5.42

For PTC patients, the blood samples of pretreatment and aftertreatment were collected under different conditions. Patients were inhibited to take levothyroxine sodium tablets or similar euthyrox drugs for at least 3 weeks before the ^131^I ablation. One month posttreatment, patients do the blood tests with routine suppressive therapy of thyroid hormone.
